# A mixed methods evaluation of *Quit for new life*, a smoking cessation initiative for women having an Aboriginal baby

**DOI:** 10.1186/s12913-023-09496-3

**Published:** 2023-05-24

**Authors:** Emilie Cameron, Jamie Bryant, Aaron Cashmore, Erin Passmore, Christopher Oldmeadow, Sarah Neill, Andrew Milat, Jo Mitchell, Nicole Gatt, Edwina Macoun, Sally J Ioannides, Carolyn Murray

**Affiliations:** 1 Health Behaviour Research Collaborative, School of Medicine and Public Health, College of Health and Wellbeing, University of Newcastle, Callaghan, NSW 2308 Australia; 2 Priority Research Centre for Health Behaviour, University of Newcastle, Callaghan, NSW 2308 Australia; 3 Hunter Medical Research Institute, New Lambton Heights, NSW 2305 Australia; 4 Population and Public Health Division, NSW Ministry of Health, St Leonards, NSW 2065 Australia; 5 Faculty of Medicine and Health, The University of Sydney School of Public Health, Sydney, NSW 2006 Australia; 6 Clinical Research Design, Information and Statistical Support, Hunter Medical Research Institute, New Lambton Heights, NSW 2305 Australia; 7 The University of New South Wales School of Medicine, UNSW, Sydney, NSW 2052 Australia; 8 Drug Health Services, Aboriginal Health Education Officer, South Western Sydney Local Health District, Sydney, NSW Australia

**Keywords:** Aboriginal, Indigenous, Pregnancy, Pregnant women, Smoking, Smoking cessation, Service delivery, Evaluation

## Abstract

**Background:**

Quit for new life (QFNL) is a smoking cessation initiative developed to support mothers of Aboriginal babies to quit smoking during pregnancy. The state-wide initiative provides support for pregnant women and their households including free nicotine replacement therapy (NRT) and follow up cessation advice. Services are also supported to implement systems-level changes and integrate QFNL into routine care. This study aimed to evaluate: (1) models of implementation of QFNL; (2) the uptake of QFNL; (3) the impact of QFNL on smoking behaviours; and (4) stakeholder perceptions of the initiative.

**Methods:**

A mixed methods study was conducted comprising semi-structured interviews and analysis of routinely collected data. Interviews were conducted with 6 clients and 35 stakeholders involved in program implementation. Data were analysed using inductive content analysis. Aboriginal Maternal and Infant Health Service Data Collection (AMDC) records for the period July 2012-June 2015 were investigated to examine how many eligible women attended a service implementing QFNL and how many women took up a QFNL support. Smoking cessation rates were compared in women attending a service offering QFNL with women attending the same service prior to the implementation of QFNL to determine program impact.

**Results:**

QFNL was implemented in 70 services located in 13 LHDs across New South Wales. Over 430 staff attended QFNL training, including 101 staff in Aboriginal-identified roles. In the period July 2012-June 2015 27% (n = 1549) of eligible women attended a service implementing QFNL and 21% (n = 320) of these were recorded as taking up a QFNL support. While stakeholders shared stories of success, no statistically significant impact of QFNL on smoking cessation rates was identified (N = 3502; Odds ratio (OR) = 1.28; 95% Confidence Interval (CI) = 0.96–1.70; p-value = 0.0905). QFNL was acceptable to both clients and stakeholders, increased awareness about smoking cessation, and gave staff resources to support clients.

**Conclusion:**

QFNL was perceived as acceptable by stakeholders and clients and provided care providers with knowledge and tangible support to offer women who presented at antenatal care as smokers, however, no statistically significant impact on rates of smoking cessation were found using the measures available.

**Supplementary Information:**

The online version contains supplementary material available at 10.1186/s12913-023-09496-3.

## Background

Through strength and resilience in the face of inequality and the disruption of culture caused by colonisation, smoking rates among pregnant Australian Aboriginal and Torres Strait Islander (hereafter respectfully referred to as Aboriginal) women have declined over time, from 50% in 2009 to 44% in 2019 [1]. However, compared to non-Aboriginal pregnant women (11% in 2019, age standardised) [1], smoking rates for pregnant Aboriginal women remain unacceptably high. Smoking is one of the most important modifiable causes of adverse pregnancy outcomes [2]. As a result of higher smoking rates, amongst other factors, Aboriginal mothers experience double the incidence of low birthweight (11.9% versus 6.4%) compared to non-Aboriginal mothers, and have higher rates of perinatal deaths (14.8 versus 8.5 per 1000 live births) and preterm deliveries (13.8% versus 8.4%)[3, 4]. The intergenerational impact of perinatal smoking is felt by the child throughout their life with increased risks of obesity, chronic conditions such as Type II diabetes and high blood pressure, and behavioural and learning disorders [5]. These impacts perpetuate disadvantage and poorer health outcomes [6].

Encouragingly, there is little difference in perinatal outcomes between Aboriginal mothers who do not smoke and the overall population of mothers in New South Wales (NSW), Australia’s most populous state [4]. There is also evidence that ceasing smoking during pregnancy reduces the risk of adverse pregnancy outcomes to levels almost the same as non-smokers [7–9]. However, the age-standardised rate of smoking cessation during pregnancy among Aboriginal mothers in 2019 was 12%, which is less than half that of non-Aboriginal mothers (25%) [1].

Despite the existence of effective smoking cessation programs for pregnant women[10], few studies have explored strategies to reduce smoking amongst pregnant Aboriginal women. A 2014 review [11] and subsequent literature search identified only three randomised controlled trials, one of which was conducted in Australia, that had examined the effectiveness of strategies to support smoking cessation amongst pregnant Indigenous women worldwide. None produced statistically significant results [12–14]. There are, however, several feasibility and exploratory studies conducted in Aboriginal communities which suggest strategies that may work to help pregnant Aboriginal women quit smoking. These include providing smoking cessation advice that is culturally appropriate and locally tailored, considering smoking in the family and community context, increasing community knowledge about smoking harm and the safety of supports to assist with quitting, providing prolonged cessation support, and midwives and doctors providing support and access to suitable forms of nicotine replacement therapy [15–17].

To support mothers of Aboriginal babies to quit smoking during pregnancy, in 2012 NSW Health developed the state-wide Quit for new life (QFNL) initiative. QFNL provides smoking cessation support to mothers of Aboriginal babies, and their household members, as part of routine antenatal and postnatal care. QFNL incorporates multiple components to influence smoking outcomes at both the patient and service levels. Support provided to mothers of Aboriginal babies includes brief cessation advice, free nicotine replacement therapy (NRT) when clinically appropriate, referral to NSW Quitline (see Table 1 for description), follow up support and self-help information. QFNL was primarily designed for implementation in Aboriginal Maternal and Infant Health Services (AMIHS) and Building Strong Foundations for Aboriginal Children, Families and Communities (BSF) services (see Table 1) across NSW but could also be applied in other settings. These services were supported to adopt and embed QFNL into usual clinical practice through an implementation handbook, periodic staff training, policy development, access to a supply of free NRT and performance monitoring and feedback. Implementation of QFNL commenced in 2013 with funding provided by the NSW Ministry of Health for a period of five years to June 2018. QFNL was implemented in 13 of the 15 local health districts (LHDs) in NSW. Reflecting NSW Health’s devolved governance structure, LHDs adapted QFNL implementation to meet local needs. At the time of program initiation, it was estimated that 56% of mothers of Aboriginal babies in NSW who smoked would receive QFNL using this implementation model.

QFNL is a large scale and complex initiative that was implemented in a real world context [18]. At the time of implementation, an evaluation plan was established to gather information about the implementation models, service delivery, outcomes and achievements, as well as management of implementation challenges. Evaluations of system-level initiatives are rarely published in the literature [19], but can provide crucial evidence about why a complex program succeeds or fails, what can be improved or optimised, and capture unintended outcomes which may become more likely as program complexity increases [20, 21].

### Aims

This study aimed to investigate:

1. models of implementation of QFNL;
2. uptake of QFNL;
3. the impact of QFNL on smoking behaviours of mothers pregnant with an Aboriginal baby; and
4. stakeholder perceptions of QFNL.

**Table 1:** Setting for implementation of QFNL.

Setting	Description
NSW Health	NSW Health is a network of local health districts, specialty networks and other health organisations that plan, manage and deliver health services for the NSW population. NSW Health is the largest health care system in Australia [22].
NSW Ministry of Health	The NSW Ministry of Health monitors and manages performance and guides the development of services and investments across the NSW Health system.
Local health district (LHD)	LHDs are responsible for providing healthcare services within a defined geographical area. Within each LHD, decisions about the provision of care and services are made locally with involvement from clinicians and the community. NSW is divided into 15 LHDs- 8 located in the Greater Sydney metropolitan area and 7 located in regional and rural areas. [23]
Aboriginal Maternal and Infant Health Service (AMIHS)	Clinics where midwives and Aboriginal Health Workers (AHWs) provide culturally safe, women-centred antenatal and postnatal care (up to 8 weeks postpartum) to mothers of Aboriginal babies [24].
Building Strong Foundations (BSF) service	Culturally appropriate early childhood health service provided by teams of child and family health nurses (CFHNs) and AHWs, for Aboriginal children from birth to school-entry age and their families [25].
Pharmacy guild of NSW	The Pharmacy guild provides information, advice and data collection to member pharmacies across NSW.
NSW Quitline (Quitline)	A free confidential telephone information and advice service designed to help smokers quit and stay quit. Aboriginal Quitline is available to Aboriginal and Torres Strait Islander people who would like to quit smoking and provides culturally sensitive advice and support.

## Methods

### Design

A mixed methods study was conducted comprising semi-structured interviews and analysis of routinely collected data.

### Setting

The study was conducted in NSW, Australia. Table 1 describes the different services and administrative levels involved.

### Semi-structured interviews

#### Interviews with key stakeholders

A series of interviews were conducted in 2015 (21 interviews) to understand how QFNL had been implemented, integrated and accepted in each LHD. Interviews were conducted with the QFNL coordinator and key staff in each of the 13 LHDs involved in implementing QFNL, as well as stakeholders from key organisations involved with QFNL. Additional interviews were conducted in 2018 (11 interviews) in three purposively selected case study LHDs [26] in order to understand the long-term processes, governance, and sustainability of QFNL. Potential interviewees were identified by the NSW Ministry of Health and invited to participate by email from the researchers. Interviewees could also nominate others to invite for interview. A semi-structured interview guide tailored to the role of the interviewee was used to guide each interview and included questions about: their role with QFNL; how QFNL had been implemented (including preparation for implementation, governance, the model used, the services involved, the support provided to staff and how uptake was monitored); how QFNL had changed routine care; key achievements and challenges; and perceptions of the appropriateness and sustainability of the QFNL model. A total of 32 interviews were conducted with 35 stakeholders. Twenty-five interviews were with a single stakeholder and 7 were joint interviews with stakeholders from the same organisation or service. Four stakeholders were interviewed at both time points. Interviews were conducted by EC by phone (27 interviews) or face-to-face (5 interviews) at the interviewee’s workplace and audio recorded with permission. Field notes were completed immediately following each interview and sent to interviewees to review.

#### QFNL clients

In 2018, interviews were conducted with QFNL clients in three LHDs, as part of a case study analysis [26]. Women were eligible to participate in an interview if they were pregnant or had given birth in the last six months; were older than 18 years; reported smoking at their first antenatal appointment; and had received any component of QFNL. Potential participants were identified using client records and were approached by staff providing their care. They were provided with information about the study either over the phone or at an appointment. Interested clients completed a consent form and provided their preferred times to be contacted. All interviews were organised and conducted by an Aboriginal woman over the phone, were audio-recorded with permission and transcribed. At the start of the interview the interviewer introduced herself as an Aboriginal woman, explained the study and confirmed consent to participate at an agreed time. A semi-structured interview guide was used to collect detailed information about their smoking; whether they wanted to quit smoking during their pregnancy; details of any support they received to quit smoking; what had and hadn’t worked to help them quit smoking; and the appropriateness of the support provided. Interviewees were provided with a $50 gift voucher as reimbursement for their time taken to participate.

#### Analysis

An inductive content analysis approach was used to analyse the data separately for staff and clients [26]. Each interview was read line by line and given a code based on an interpretation of the content. Coding was conducted by one researcher and checked by another member of the research team. Codes were refined until agreement was achieved. A coding matrix, grouping codes into categories sharing a commonality, was developed following analysis of the first 3 interviews. Codes in subsequent interviews were assigned to this coding matrix with any additional codes that did not fit the matrix added to ensure all data was captured. The robustness of conclusions was tested by comparing codes within each interview and between interviews. Themes were developed across categories to allow for interpretation of codes and help structure the presentation of the study results.

### Routinely collected data

#### Data source

The Aboriginal Maternal and Infant Health Service Data collection (AMDC) holds records of births in NSW for which the baby or the baby’s mother is recorded as Aboriginal. AMDC data were available for 11 of the 13 LHDs implementing QFNL for the period July 2012-June 2015. Two LHDs that implemented QFNL used a different data collection program that did not contribute data to the AMDC. The following routinely collected variables were obtained for the study: month and year of birthing; Aboriginal status of the mother; age of mother; socio-economic indexes for statistical local Area (SEIFA) of residence, model of antenatal care received; date of first antenatal care visit; whether an AMIHS service was attended; LHD of birthing hospital; uptake of core QFNL supports (NRT, Quitline or follow up support); smoking status in the first (“*Are you a smoker?”*) and second (“*Have you smoked during the second half of pregnancy?”)* half of pregnancy (yes, no, not stated); and number of cigarettes smoked per day in the first half and second half of pregnancy. The sample for analysis included all women eligible for QFNL. That is those giving birth to an Aboriginal baby who smoked in the first half of pregnancy and had at least one antenatal care visit.

#### Uptake

The number of eligible women in the sample who attended a service implementing QFNL was counted. Among these, uptake was defined as the number recorded as taking up one or more of the core QFNL supports (NRT product or a voucher for NRT, referral to Quitline or referral for follow up support). The AMDC data for uptake was entered into a free text box created specifcally for capturing implementation of QFNL and interpreted according to the rules in additional file 1. LHDs also maintained separate records of all clients who took up a QFNL support during pregnancy and any NRT that was supplied. These data were kept by those involved with QFNL and reported directly to the NSW Ministry of Health each quarter. The uptake of core supports was compared between these two data sources to check the accuracy of AMDC records.

#### Impact

The main outcome measure used was smoking cessation. Women were considered to have ceased smoking if they were recorded as smoking in the first half of pregnancy and not smoking in the second half. This measure is used in National reporting [27] and is a key performance indicator for LHDs [28]. As a measure of the impact of QFNL, smoking cessation rates were compared between eligible women attending a service offering QFNL after the implementation of QFNL had begun (post-QFNL), with women attending the same service prior to the implementation of QFNL (pre-QFNL). In this way each service acted as its own control to account for the fact that participation in QFNL was not randomised. The change in the number of cigarettes smoked per day in the first to the second half of pregnancy was calculated in the pre and post-QFNL groups as a secondary measure of impact. Smoking cessation and number of cigarettes smoked per day were also examined in the post-QFNL group in those recorded as taking up a QFNL support and those who didn’t take up a support.

#### Analysis

AMDC records were analysed using SAS Version 9.42. Characteristics of all the eligible women included in the sample are presented. Characteristics associated with taking up a QFNL support were explored using a logistic regression model adjusted for LHD of service and year of baby’s birth and including the variables: Aboriginal status of mother, maternal age, socio-economic status (based on SEIFA [29] of residential area) and number of antenatal care visits. Similar models were constructed to explore the measures of impact (Smoking cessation and change in number of cigarettes smoked per day) in the pre and post QFNL groups and those in the post-QFNL group taking up a QFNL support or not. Clustering data at the site level was not available for QFNL sites, so robust Hubert-White standard errors were used for all models.

## Results

### Participants

Interviews were conducted with 6 clients and 35 stakeholders. Stakeholders included QFNL coordinators in all 13 LHDs implementing QFNL, smoking care advisors, health promotion managers, midwives, Aboriginal Health Workers, service managers. and representatives from the NSW Ministry of Health, the Aboriginal Health and Medical Research Council of NSW and NSW Quitline. Interviews ranged from 20 to 90 mins duration.

### QFNL implementation

Most LHDs commenced implementation of QFNL over a one year period between August 2013 and August 2014. One LHD commenced earlier, in January 2013 and two later, in October 2014 and January 2015. Overall, QFNL was implemented in 13 of 15 LHDs and 70 individual services. The number of services offering QFNL varied across LHDs from one site to 16, reflecting the LHD size, number of services and local implementation model. Implementation occurred mostly through AMIHS and BSF postnatal services, however, some LHDs also implemented QFNL in hospital-based antenatal care services, community health settings or non-government community organisations working with Aboriginal families.

Each LHD had a QFNL coordinator who managed implementation. A coordinator network was set up to enable the sharing of ideas and documents. State-wide training on the QFNL supports, policies and procedures and motivational interviewing techniques was provided in person by a state-based trainer at local one day workshops. A total of 439 LHD staff attended training between January 2013 and April 2015, including midwives (n = 114), child and family health nurses (n = 87), health promotion staff (n = 28) and tobacco, drug and alcohol workers (n = 20). 23% of those trained were employed in Aboriginal-identified roles including Aboriginal Health Workers (n = 30), Aboriginal Education Officers (n = 52) or in other Aboriginal health roles (n = 21). Additional training on using yarning (culturally appropriate and purposeful conversation[30]) to support Aboriginal women to quit was offered online and some LHDs organised their own training programs.

To support implementation, relationships were established with NSW Quitline and the NSW Pharmacy Guild (see Table 1). NSW Quitline offers a pregnancy specific call schedule and clients can request they speak with an Aboriginal advisor through the Aboriginal Quitline service. Through QFNL clients could be provided with NRT either directly or via a voucher to be redeemed at a pharmacy after an assessment that it was clinically appropriate. To aid in the provision of NRT, an NRT policy, specific for QFNL and based on management guidelines [31], was developed by the NSW Ministry of Health. The pharmacy guild helped establish the NRT voucher system which was needed in LHDs where policies prevented direct provision by some staff or where QFNL was not provided face-to-face. Some LHDs also used carbon monoxide monitors or incentives such as baby bibs to engage clients.

Three models of QFNL delivery were identified. The advantages and disadvantages of each model, as identified by participants, are outlined in Table 2.

1) Referral model (implemented by 10 LHDs). In this model, clinic staff referred women identified as smokers to a dedicated smoking care advisor to receive cessation support. There were differences by LHD in the degree to which QFNL supports were provided by the clinic staff prior to referral, the type and number of staff hired for the smoking care advisor role and the type of follow-up care provided. For example, in one geographically large LHD a centralised team provided cessation care primarily over the phone. They offered QFNL supports after clinic staff initiated the discussion about quitting and organised referral to QFNL. In another LHD, clinic staff initiated discussions and offered the QFNL supports including referral to an Aboriginal smoking care advisor for family focused home visits. In other LHDs the smoking care advisor was co-located with maternity services and available to see women following their regular antenatal appointment.

2) Capacity building model (implemented by 1 LHD). In this model, QFNL was integrated into the role of all staff providing antenatal and postnatal care. Women attending an appointment were provided behavioural smoking assessments and offered QFNL supports as appropriate. Cessation support was provided during regular appointments, with additional appointments available if extra support was required. QFNL support officers assisted services in the initial stages of implementation, e.g. by coordinating training and provision of resources to services, identifying barriers and addressing underperformance through staff coaching. These roles were phased out as QFNL became embedded into routine care.

3) Direct service provision model (implemented by 2 LHDs). This model combines elements of both the referral and capacity building models. A smoking care advisor role was integrated into the role of an existing staff member. Clinic staff provided brief advice and offered referrals to the NSW Quitline. They then referred interested clients to the local staff member to deliver follow-up care and provide NRT if appropriate.

**Table 2:** Advantages and disadvantages identified by stakeholders for each QFNL implementation model

Model	Advantages	Disadvantages
Referral	• Less burden on clinic staff • More time available to address smoking • Skilled, confident smoking care advisor addresses smoking	• Relies on funding for smoking care advisor position • Affected by staff turnover • Limited support for women who decline referral to smoking care advisor
Capacity Building	• Sustainable beyond funding period • Large number of staff can deliver support • Smoking addressed often by someone with existing relationship with client	• Staff have limited time to address smoking • Staff may lack confidence or not see it as their role to address smoking • Constant need to train and update all staff
Direct Service Provision	• Utilises local resources • Less burden on clinic staff • Skilled, confident staff address smoking	• Time burden on staff providing care • Affected by staffing and skill gaps • Limited support for women who decline referral to smoking care advisor

### Uptake of QFNL

Between July 2012 and June 2015 there were 14,113 births in NSW for which the baby or the baby’s mother was recorded as Aboriginal and the woman received antenatal care in an LHD that implemented QFNL and contributed data to the AMDC (n = 11 LHDs). Of these 5798 (41%) were recorded as smoking in the first half of pregnancy and therefore eligible for QFNL. The characteristics of these women are shown in Table 3. Over this period 27% (n = 1549) of eligible women attended a service implementing QFNL. This value rose from 1.4% (n = 27) between July 2012 and June 2013 to 53% (n = 1030) between July 2014 and June 2015.

**Table 3:** Characteristics of women eligible for QFNL recorded in the AMDC between July 2012 and June 2015 (N = 5798)

Characteristic	Category	Mean (SD)
Maternal age (years)	-	26 (6)
Number of antenatal care visits	-	9 (10)
**Characteristic**	**Category**	**N (%)**
Indigenous status of mother	Aboriginal or Torres Strait Islander	4013 (69%)
	Neither	1778 (31%)
First antenatal care visit	Before 20 weeks gestation	4239 (73%)
	After 20 weeks gestation	1559 (27%)
Type of service attended	AMIHS	2982 (51%)
	Non-AMIHS QFNL service	520 (9.0%)
	Non AMIHS	2296 (40%)
Socio economic status (SEIFA)	1–5 (Most disadvantaged)	5130 (89%)
	6–10 (Least disadvantaged)	630 (11%)

AMDC records indicate 21% (n = 320) of eligible women attending a service implementing QFNL (n = 1536) took up a core QFNL support. More women took up offers of follow-up support (190; 12%) and NRT (168; 11%) than Quitline referral (136; 8.8%). There were no significant factors associated with taking up a QFNL support compared to those who didn’t take up a support. LHD records suggest that uptake may be higher, with 54% of eligible women recorded as accepting a core QFNL support. This highlights a limitation with the AMDC dataset suggesting that data was not recorded well in the free text box created to collect data for the initiative.

### Impact of QFNL on smoking

To examine the impact of QFNL on smoking rates, smoking cessation was compared in those who attended a service prior to the implementation of QFNL (pre-QFNL; n = 1953) with those who attended the same service after the implementation of QFNL had begun (post-QFNL; n = 1549). In the pre-QFNL group 19% (n = 374) ceased smoking compared to 18% (n = 276) in the post-QFNL group. Generalized linear modelling found no significant difference in smoking cessation between the pre-QFNL and post-QFNL groups adjusting for potential cofounders (Table 4). Similarly, there was no difference in smoking cessation between those in the post-QFNL group who took up a QFNL support and those who didn’t (19% and 18% respectively; N = 1549; Adjusted Odds ratio (OR) = 1.09; 95% CI = 0.84–1.42; p-value = 0.49).

Overall, those in the post-QFNL group smoked 0.81 fewer cigarettes per day in the second half of pregnancy compared to those in the pre-QFNL group who smoked 0.73 fewer per day. This difference was not significant when adjusting for potential cofounders (N = 3502; adjusted OR = 0.31; 95% CI =-0.35-0.96; p- value = 0.35). Those in the post-QFNL group who took up a QFNL support smoked 1.20 fewer cigarettes per day in the second half of pregnancy compared to 0.71 fewer for those not taking up a QFNL support. Again, this difference was not significant after adjusting for potential cofounders (N = 1549; Adjusted OR=-0.16; 95% CI=-0.98-0.65; p-value = 0.68).

**Table 4:** Factors associated with ceasing smoking before the second half of pregnancy (N = 3502)

Predictor	Summary	Ceased smoking		Adjusted OR (95% CI) †	p-value
		Yes	No		
Group	Pre-QFNL implementation	374 (19%)	1579 (81%)	ref	
	Post-QFNL implementation	279 (18%)	1273 (82%)	1.28 (0.96, 1.70)	0.0905
Aboriginal status of mother	Aboriginal	466 (18%)	2159 (82%)	1.16 (0.96, 1.40)	0.1290
	Non-Aboriginal	184 (21%)	693 (79%)	ref	
SEIFA	1–5 (Most disadvantaged)	559 (18%)	2581 (82%)	1.10 (0.83, 1.46)	0.4871
	6–10 (Least disadvantaged)	86 (25%)	257 (75%)	ref	
Maternal age	Mean (SD)	25 (6)	26 (6)	1.01 (1.00, 1.03)	0.1277
No of antenatal care visits	Mean (SD)	10 (8)	9 (10)	1.00 (0.99, 1.00)	0.2600

Exclusions: Women who did not receive antenatal care, did not smoke in the first half of pregnancy, received care in an LHD not included in AMDC data or did not attend a QFNL service

†Adjusted for LHD, year of baby’s birth, and all covariates presented in the table

### Stakeholder and client perceptions of QFNL

Broad themes emerged during interviews with stakeholders and clients around the appropriateness of QFNL, achievements recognised, enablers of success and challenges experienced. Table 5 provides illustrative quotes around those themes.

**Table 5:** Quotes from stakeholders and clients illustrating the major themes in perceptions of QFNL.

Theme	Illustrative quotes
**Stakeholders**	
Appropriateness of model	It was a really good way, I think, of introducing the fact that we really do want to try and help them to stop smoking for the benefit of their babies and themselves. (Midwife)
	Smoking does come up a few times in their antenatal and postnatal checks already. So having Quit for new life helped you to expand on that rather than just asking a question. (Aboriginal Health Worker)
Achievements	One of the key things it’s done for me is that it’s increased my prioritising of addressing smoking within pregnancy. (Midwife)
	We had success with a whole family quitting and saving $110 a day. (QFNL coordinator)
	So we noticed that women were adopting the breastfeed, feed first, have your cigarette after; smoking outside, not smoking in the car, wearing of a smoking shirt, reducing co-sleeping when smoking. (Service manager)
Enablers	We have really tried to promote the Aboriginal health workers taking the lead, they’re the ones that have the best cultural knowledge and skills for engaging with the mums and talking to them in a way that can make them feel comfortable when they’re opening up about their smoking journey. (QFNL coordinator)
	But I think the best thing about the program is yeah, the fact that the NRT was available to the woman, the partner and anyone else that’s in the household. (Smoking care advisor)
Challenges	Behavioural support is not yet fully embedded in appointments as it is time consuming in an environment that is already stretched. (QFNL coordinator)
	QFNL addresses a health issue which women may not see as a problem. (QFNL coordinator)
	We are constantly trying to keep up with changes in staff and organising training. (QFNL coordinator)
	It is difficult to follow-up with clients because of the rural area and poor mobile coverage (Smoking care advisor)
	Measures on changes in smoking rates doesn’t sensitively capture women who engaged in quit periods during pregnancy. (QFNL coordinator)
**Clients**	
Appropriateness of model	It made me realise a lot of things - the reasons I had to stop…The midwives were pushing for me to do it, because they know the effects it has, I guess. (Client A)
	I have a worker and she comes and meets me and we have chats. I get the [NRT] vouchers off her. Whenever I’m needing her, she’s available for me to contact… I left the program at one point and then I was welcome to come back at any time. It was really good. (Client B)
	Yeah, about 10 out of 10 probably, because they just offered a whole range of support for me. (Client C)
Achievements	I’ve been trying for months, before getting pregnant, to quit smoking and I just had no sort of motivation or anything like that. So, getting into the program had that sort of motivation. (Client D)
	I had two attempts. I think the second attempt - I was on it [NRT] for about maybe a month and a half, two months, and that’s been since I’ve quit. (Client C)
	We smoked inside, but now that bub is here we smoke outside. (Client E)
Enablers	Well, just for bub. Yeah, that was my main reason. (Client E)
	She [smoking care advisor] has actually been a smoker. So that kind of helped. (Client B)
Challenges	I tried the gum but it was disgusting so I didn’t use it. (Client F)
	Really, really pleased [with the support received]. I think it was just me, more that I needed to more want to quit. (Client E)
	It was a bit confronting. I guess it’s an addiction. It’s kind of hard to talk about it sometimes (Client A)

#### Key stakeholders

Most stakeholders interviewed considered QFNL appropriate for addressing smoking in the target group. They reported that having QFNL led to increased awareness about smoking cessation among pregnant women, Aboriginal community groups, families, health professionals and LHD management. The training provided increased the knowledge and awareness of staff, so they were comfortable to address smoking in a culturally sensitive way. Building the capacity of Aboriginal Health workers to provide cessation care and take a leadership role in QFNL was seen as particularly valuable.

Stakeholders reported that staff knowing what cessation support they could offer and having resources available, in particular the provision of free NRT and policies around its use, were helpful. Carbon monoxide monitors were seen as a good tool for engaging clients. Many stakeholders reported seeing positive changes in clients such as accepting referrals for support, adopting safer practices for the baby, quitting, attempting to quit, reducing the number of cigarettes smoked and returning for subsequent pregnancies having remained smoke-free, or willing to try quitting again.

Stakeholders perceived that having the support of management, appropriate governance structures, working closely with services and having processes to feedback progress contributed to successful implementation. Under all implementation models, clinic staff initiated the discussion about cessation and needed to have the knowledge, confidence, support and time to do this in an environment with many competing priorities. However, staff turnover and short-staffed clinics often made it difficult to maintain training and could lead to gaps in the delivery of QFNL. Stakeholders reported some challenges with data reporting, expressing concerns about the content, accuracy and monitoring of the data.

Some staff implementing QFNL reported challenges with engaging clients to take up QFNL supports. These staff felt that some clients did not consider smoking a problem particularly when other difficulties in their lives were more of a priority. It was sometimes difficult to schedule extra appointments or maintain phone contact. Stakeholders appreciated being able to provide supports to household members and suggested that the most successful quit attempts occurred when household members were involved.

#### Clients

Clients reported different levels of engagement with QFNL and various degrees of success with quitting smoking. All clients interviewed were satisfied with the support that they received. They thought that smoking should be addressed during the antenatal period when the health of the baby was a big motivator. Having antenatal staff address smoking made them realise that quitting was important and that they had support to quit.

The availability of free NRT and support around its use was well received, particularly the ability to trial different types of NRT to find the one that suited their needs, circumstances and preferences. Some clients blamed themselves rather than the support received for not being able to quit. Factors that assisted with engaging clients included the clinic staff or smoking care advisor being Aboriginal themselves or having lived experience of quitting, taking the time to build a rapport with the client and being easily accessible.

## Discussion

There is currently no evidence for effective strategies to reduce smoking amongst pregnant Aboriginal women [11, 13]. QFNL is a novel approach to cessation for pregnant mothers of Aboriginal babies that incorporates promising elements identified in feasibility studies. This mixed methods evaluation of QFNL represents a unique opportunity to evaluate the effectiveness of a complex initiative in a real-world context by combining process and impact measures to enable a robust assessment of the acceptability of the initiative, extent to which it was implemented as planned, and its impact on smoking behaviours.

QFNL was implemented at 70 services across 13 LHDs in NSW under 3 implementation models: capacity building, referral system and direct service provision. Aboriginal people were involved in the delivery and implementation of the model. A recent survey highlighted the positive impact of having Aboriginal health workers involved with patient care, including among maternity patients [32]. Stakeholders considered the QFNL model to be appropriate to address smoking in the target group but noted several implementation challenges including engaging clients, maintaining staff training, gaining management support and data reporting.

Achievements in implementation identified by stakeholders included seeing positive changes in clients, increased staff knowledge and awareness of the need to address smoking, the inclusion of household members and having tangible supports, such as NRT, available to offer clients. Clients who received QFNL reported that it was appropriate to address smoking during pregnancy since the health of the baby was the main motivation to change their behaviour. Interviews revealed that they were satisfied with the support they received, especially the availability of NRT and ability to try different types to find one that worked; and talking with an Aboriginal woman who understood their circumstances. This finding is consistent with the findings of a recent survey of women receiving QFNL in one LHD [33]. A high proportion of women in that study accepted cessation support, with most accepting NRT. They identified barriers to accepting support and suggest that strengthening the role of Aboriginal health workers, increased support in the use of NRT, increasing offers of support and improving the cultural appropriateness of strategies to engage women will improve acceptance.

While QFNL was acceptable to both clients and stakeholders, analysis of routinely collected AMDC data suggests QFNL did not have a significant impact on rates of smoking cessation during the three years examined. QFNL supports were selected for their promising results in feasibility and exploratory studies as well as success in general populations [15–17]. However, they may not be successful in a real world setting with competing priorities and stressors. Alternatively, QFNL may have an impact which was not detected by the measures and timeframes used in the evaluation (smoking cessation and change in amount smoked per day from the first to second half of pregnancy). Smoking cessation can be a difficult process with multiple quit attempts often required before quitting is successful [34, 35]. There were stories shared during interviews about changes to smoking behaviour and women having multiple quit attempts and a survey of QFNL clients in 1 LHD found that 63% had quit for 1 or more days and 35% had abstained from smoking for 1 month or more [33]. Quit attempts were not consistently collected for monitoring of QFNL.

Other evaluations of programs addressing smoking have had mixed success. Many studies in antenatal or indigenous settings have low recruitment and high rates of drop out [36–38]. However, a recent systematic review [39] and meta-analysis of implementation strategies to support health professionals to address smoking in the antenatal period indicated that interventions led to an increase in women being asked, assessed, advised and assisted to arrange support for smoking and that strategies to improve care practices did increase the rate of cessation. They highlighted the need for multiple strategies to be used together particularly those targeting specific barriers.

NSW Health is using the findings of this study, together with other evidence, to improve smoking cessation support for pregnant Aboriginal women. Targeted strategies include the ongoing delivery of QFNL, offering the *Yarning About Quitting* program [40], and providing funding for Aboriginal Community Controlled Health Services to deliver free NRT to their midwifery clients (when clinically appropriate). Universal approaches are also being implemented. These include enhancing clinical software to enable digital referrals to NSW Quitline, establishing clinical standards for treating smoking before, during and following pregnancy, training maternity staff in cessation care, and establishing an antenatal smoking cessation performance measure for LHDs. NSW Health is also supporting the national Safer Baby Bundle initiative, which aims to prevent still birth. Increasing smoking cessation during pregnancy forms one of five key elements of the program, and includes tailoring for priority populations, including pregnant Aboriginal women.

### Limitations

Potential limitations should be considered when interpreting the findings of this evaluation. The number of clients interviewed was small and recruited via convenience sampling, therefore the results may not reflect the views of those who are difficult to reach or who did not engage with QFNL. As participation in QFNL was not randomised we could not control for differences in potentially confounding factors, such as education or employment, between women attending different service types. However, confining the analysis to QFNL services meant that each service acted as its own control, therefore limiting the effect of these potential biases. Data reporting was a common problem encountered by stakeholders and reflected in the results here. QFNL specific data on the uptake of supports was captured using a free text box. This text was not always entered by the person offering the supports and, in some cases, did not specify which support was taken up. Comparison with LHD maintained records suggests that some supports taken up by clients were not recorded in the AMDC. No data were captured on offers of support, whether the supports taken up were used or what is happening during the postpartum period. The measure of impact is a commonly used indicator of smoking cessation used in National reporting [27]. However, it only considers instances of successful smoking cessation prior to the second half of pregnancy, which leaves relatively little time for QFNL to have a measured effect. Similarly, the analysis of the number of cigarettes smoked per day was for the change from first to second half of pregnancy.

## Conclusions

QFNL is a smoking cessation program designed to help mothers of Aboriginal babies quit smoking while pregnant. QFNL was implemented differently in LHDs across NSW considering local resources, conditions and capacities. QFNL was perceived as acceptable by stakeholders and clients and gave care providers knowledge and tangible supports to offer women who presented at antenatal care as smokers, however no significant impact on rates of smoking cessation were found using the measures provided for the evaluation.

## Electronic supplementary material

Below is the link to the electronic supplementary material.

Supplementary Material 1

## Data Availability

Restrictions apply to the availability of AMDC data. Requests for access to these data can be directed to the AMDC custodian, NSW Health. All relevant qualitative data are presented in this paper. Additional qualitative data could be available upon reasonable request to the corresponding author.

## Abbreviations

AMDC: Aboriginal Maternal and Infant Health Service Data Collection.
AMIHS: Aboriginal Maternal and Infant Health Service.
BSF: Building Strong Foundations for Aboriginal Children, Families and Communities.
CI: Confidence Interval.
LHD: Local Health District.
NRT: Nicotine Replacement Therapy.
NSW: New South Wales.
OR: Odds Ratio.
QFNL: Quit For New Life.
SD: Standard deviation.
SEIFA: Socio-Economic Indexes for Areas.
