# Supplementary material for: A mixed methods evaluation of Quit for new life, a smoking cessation initiative for women having an Aboriginal baby
Source: BMC Health Serv Res. 2023 May 24;23:532. doi: 10.1186/s12913-023-09496-3 (PMC10210382; doi:10.1186/s12913-023-09496-3)
Supplement: Supplementary file 1 — Supplementary Material 1 [file 12913_2023_9496_MOESM1_ESM.docx]

**Additional files**

**Additional file 1**: Rules used to define QFNL uptake from data entered in a free text box field.

| Textbox entry | Coded as* |
| --- | --- |
| Any descriptions of NRT:  *e.g. NRT, nicotine replacement, lozenge, inhaler* | QFNL, NRT |
| Any descriptions of referral or follow-up:  E.g. *follow-up, quit referral, QFNL advisor, referred for QFNL, counselling, quit smoking clinic, AMIHS D&A for smoking cessation support.* | QFNL, follow-up |
| Any description of telephone based support line  *e.g. Quitline, hotline* | QFNL, Quitline |
| If intervention refused:  e.g. *QFNL offered and declined, NRT refused* | No |
| If intervention offered, with no indication of whether taken up or refused:  e.g. *NRT offered* | QFNL, intervention name |
| General description of QFNL,  e.g. *QFNL, quit program, smoking cessation, QUIT* | QFNL |

* The intervention taken up is named where possible otherwise only the QFNL code is used.
